# Is surgical subspecialization associated with hand grip strength and manual dexterity? A cross-sectional study

**DOI:** 10.1016/j.amsu.2021.103159

**Published:** 2021-12-07

**Authors:** Reickly D.N. Constansia, Judith E.K.R. Hentzen, Carlijn I. Buis, Joost M. Klaase, Vincent E. de Meijer, Mark Meerdink

**Affiliations:** University Medical Center Groningen, Department of Surgery, Groningen, the Netherlands

**Keywords:** Hand grip strength, Manual dexterity, Academic surgery

## Abstract

**Background:**

The aim of this study was to compare hand grip strength (HGS) and manual dexterity of academic, subspecialized surgeons.

**Methods:**

A single-center cross-sectional study was performed among 61 surgeons. HGS was analysed with a hand dynamometer and manual dexterity was extensively analysed with a Purdue Pegboard Test. Correlations between HGS and manual dexterity and specific characteristics of the surgeons were analysed using Pearson's correlation coefficient (*r*).

**Results:**

HGS and manual dexterity were comparable between surgeons from different specialities. HGS was positively correlated with male gender (*r* = 0.59, *p* < 0.001) and hand glove size (*r* = 0.61, *p* < 0.001), whereas manual dexterity was negatively correlated with male gender (*r* = −0.35, *p* = 0.006), age (*r* = −0.39, = 0.002), and hand glove size (*r* = −0.46, *p* < 0.001).

**Conclusions:**

Surgical subspecialization was not correlated with HGS or manual dexterity. Male surgeons have greater HGS, whereas female surgeons have better manual dexterity. Manual dexterity is also correlated with age, showing better scores for younger surgeons.

## Highlights

• Hand grip strength (HGS) and manual dexterity are crucial surgical skills.
• HGS and manual dexterity do not differ between surgeons of different specialities.
• Male surgeons have greater HGS, whereas female surgeons have better manual dexterity.
• Manual dexterity is correlated with age, showing better scores for younger surgeons.

## 1 Introduction

Great hand grip strength (HGS) and excellent manual dexterity are two of the various skills required for surgeons to perform complex surgical procedures. Such procedures usually require plenty of standing, stamina and limb movement. Hence, maintaining good physical condition and strength is crucial to ensure optimal performance during surgery. In their systematic review, Bohannon et al. showed that HGS in adults can be used as a biomarker for the overall strength, physical function and health status [1]. Manual dexterity is also important to minimise surgical damage to delicate body tissues and to guarantee patient safety. Manual dexterity is the ability to make coordinated hand and finger movements to grasp and manipulate objects of all shapes and sizes. Manual dexterity includes muscular, skeletal and neurological functions to produce small and precise movements, such as handwriting, playing a musical instrument or, in our case, safely perform surgical procedures [2].

Although a great HGS and outstanding manual dexterity are important for all surgeons, it might be argued that surgical differentiation has led to differences in both skills among surgeons of various surgical specialities [3]. For instance, on the one hand, orthopaedic trauma surgeons regularly perform surgical procedures on fractured bony structures while using their strength and relatively heavy surgical tools to perform anatomic repositioning and fixation. On the other hand, during vascular, oncological and gastro-intestinal surgeries, precisely coordinated movements of the hands are essential to create surgical anastomoses and perform resections and revascularisations of small structures.

Previous studies have shown that HGS is influenced by multiple factors such as gender, age, muscle mass, and occupation [[4], [5], [6], [7]]. Similarly to HGS manual dexterity is also influenced by various factors. Factors which have been shown to affect manual dexterity are age, gender, hand size [[8], [9], [10]]. A study examining manual dexterity of surgeons versus physicians showed that surgeons performed better than physicians [11].

To the best of our knowledge, no previous scientific research has compared HGS and manual dexterity between various surgical specialities. We therefore hypothesized that HGS would be greater for orthopaedic trauma surgeons, whereas paediatric, vascular, oncological and gastro-intestinal surgeons would have greater manual dexterity than that of their colleagues. If differences are found between the HGS and manual dexterity of academically differentiated surgeons surgical residents might take their HGS and manual dexterity in to account when choosing a surgical subspecialization.

Therefore, the aim of this study is to compare HGS and manual dexterity among academic, subspecialized male and female surgeons.

## 2 Material and methods

### 2.1 Design, setting and participants

A cross-sectional study was performed at the surgical departments of University Medical Center Groningen, Groningen, the Netherlands, between December 2019 and January 2020. All surgeons working at the surgery department of the University Medical Center Groningen were eligible to participate in this study. Forty-seven male and fifteen female surgeons were identified. Both the HGS and manual dexterity of vascular, orthopaedic trauma, paediatric, gastro-intestinal, oncological and hepatopancreatobiliary surgeons were assessed. No ethical approval was required to perform this study. This study has been registered in the Research Registry database the identifying number is: researchregistry7251.

### 2.2 Baseline characteristics and confounding factors

Baseline characteristics and possible confounding factors for HGS or manual dexterity were prospectively collected from all the participating surgeons, including gender, age, surgical speciality, years of experience as a surgeon, dominant hand and hand glove size. These confounders were identified from the current scientific literature [[4], [5], [6]]. To avoid confounding by sleep deprivation, testing of HGS and manual dexterity was performed only if the surgeon was not on call the preceding night [7].

### 2.3 Measurements

#### 2.3.1 Hand grip strength

HGS was measured to the nearest kilogram using a calibrated hydraulic hand dynamometer (Model no. 563213; Sammons Preston, USA) in a standardized setup. Surgeons were seated (single in a room) in a standard-height chair with full back support. Both elbows were flexed to 90° without rotation, with their wrists in a neutral position and their forearms supported on a table. After a try-out to get accommodated with the apparatus, all the participants were instructed to squeeze the dynamometer with the maximum force that they can apply, starting with their dominant hand followed by their non-dominant hand. HGS was measured three times for both hands over 30 s intervals to prevent fatigue. The maximum value among the three measurements was recorded for both hands, and the highest value of the dominant hand was used in this study [12,13].

#### 2.3.2 Manual dexterity

Manual dexterity was extensively evaluated using a Purdue Pegboard Test (PPT; Model no. 32020, J.A. Preston Corporation, USA). This test is a neuro-physiological test of manual dexterity and bi-manual coordination and is recommended as a validated and reliable test for assessing manual dexterity in health care professionals [2]. The pegboard consists of a board with two parallel rows containing 25 holes, into which cylindrical metal pegs are placed by the participant. The manual dexterity of each surgeon was measured using the standardized method described in the PPT instruction manual [3].

During the measurements, the surgeons were seated (single in a room) in front of the pegboard. The manual dexterity test involved a total of four trials. During the first three trials, the surgeons had to place as many pins as possible in the pegboard in a time interval of 30 s using their dominant hand, non-dominant hand and finally both hands simultaneously. The score for each trial was the number of pins placed in 30 s.

The final trial consisted of assembling pins, collars and washers using bi-manual manipulation. The surgeons were required to insert a pin into the pegboard using their dominant hand, followed by placing a washer over the pin with their non-dominant hand and then placing a collar over the washer using their dominant hand. To complete the assembly, they placed a second washer over the pin using their non-dominant hand. This task was repeated for 60 s, and the score for the trial was considered the number of pins, collars and washers placed in 60 s. The sum of the scores of all four trials represented the manual dexterity of each surgeon.

### 2.4 Primary and secondary outcomes

The primary outcome was HGS and manual dexterity for six different surgical specialities (i.e. vascular, orthopaedic trauma, paediatric, gastro-intestinal, oncological and hepatopancreatobiliary surgery). The secondary outcomes included the influence of gender, age, years of experience as a surgeon and hand glove size on HGS and manual dexterity.

### 2.5 Data analysis

All statistical analyses were performed using IBM SPSS Statistics version 24.0 (IBM Corp., Armonk, NY, USA). All *p*-values lower than 0.05 were considered statistically significant. Quantitative variables are presented as a mean ± standard deviation (SD) or as a percentage. For hypothesis testing between groups, Student's *t*-test was used for continuous variables following a normal distribution, and the Mann–Whitney *U* test was used for variables without a normal distribution. Correlations between HGS and manual dexterity and gender, age, surgical specialty, years of experience as a surgeon, hand glove size and dominant hand were analysed using Pearson's correlation coefficient (*r*). Logistic regression was performed to examine the effect of gender and hand glove size on HGS and manual dexterity.

## 3 Results

### 3.1 Baseline characteristics

In total, 61 out of 62 eligible surgeons (98%) participated in this study. One surgeon refused to participate without providing a reason to withdraw. Table 1 outlines the baseline characteristics of the entire cohort of surgeons according to gender, as well as the correlation between these characteristics and HGS and manual dexterity. Male surgeons, on average, were found to have a larger hand glove size than that of female surgeons (*p* < 0.001). Other baseline characteristics were similar between the two groups.

**Table 1:** Baseline characteristics according to gender and correlations between HGS and manual dexterity scores.

Gender			Correlation with HGS		Correlation with dexterity	
			r	p	r	p
	Male (*N* = 46, 75%)	Female (*N* = 15, 25%)	0.59	<0.001	−0.35	0.006
Age in years	45	42	−0.14	0.91	−0.39	0.002
***30–39 (n = 24, 39%)***	16	8				
***40–49 (n = 22, 36%)***	17	5				
***50–59 (n = 11, 18%)***	10	1				
***>60 (n = 4, 7%)***	3	1				
*Sub-specialty*			−0.20	0.12	0.07	0.61
***Vascular surgery (n = 8, 13%)***	7	1				
***Orthopaedic trauma surgery (n = 10, 16%)***	9	1				
***Paediatric surgery (n = 8, 13%)***	4	4				
***Gastro-intestinal surgery (n = 12, 20%)***	8	4				
***Oncological surgery (n = 10, 16%)***	7	3				
***Hepatopancreatobiliary surgery (n = 13, 21%)***	11	2				
*Years active as a surgeon*			−0.53	0.69	−0.38	0.003
***0–5***	16	6				
***5–10***	9	4				
***10–15***	8	3				
***15–20***	5	1				
***>20***	8	1				
*Hand glove size*			0.61	<0.001	−0.46	<0.001
***6.0***	0	2				
***6.5–7.0***	4	11				
***7.5–8.0***	38	2				
***8.5***	4	0				
*Dominant hand*			0.24	0.06	0.02	0.88
***Right***	44	14				
***Left***	2	1				

### 3.2 Hand grip strength

**Fig. 1:**
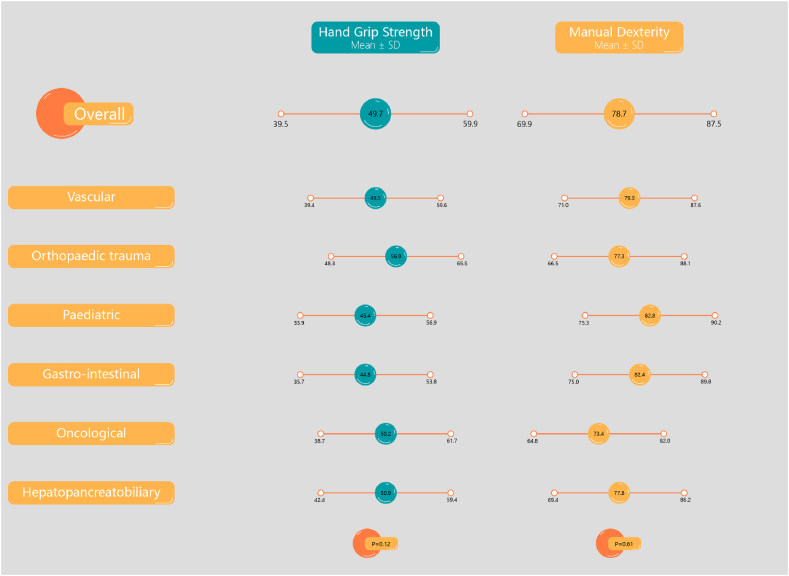
Hand grip strength and manual dexterity per surgical per surgical sub-specialty.

No significant correlation was found between HGS and surgical sub-specialty, age or years active as a surgeon (Fig. 1, Table 1). On the other hand, HGS was positively correlated with male gender (Fig. 2A) and an increased hand glove size, with the greatest strength for participants with size 8 (Fig. 2B). Multi-variate logistic regression showed that both hand glove size (*p* = 0.16) and gender (*p* = 0.66) were not significantly associated with HGS.

**Fig. 2A:**
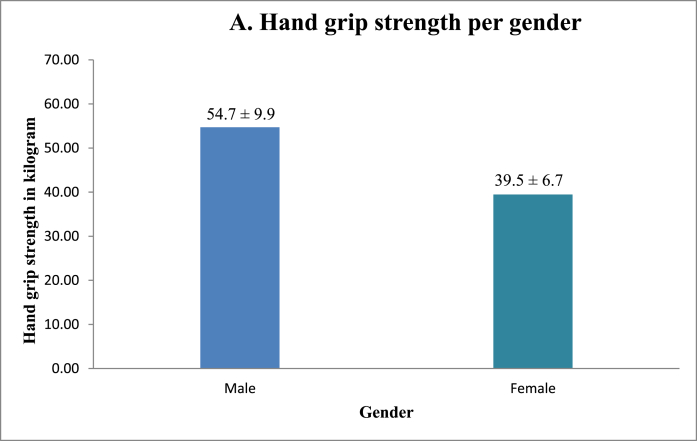
Hand grip strength per gender.

**Fig. 2B:**
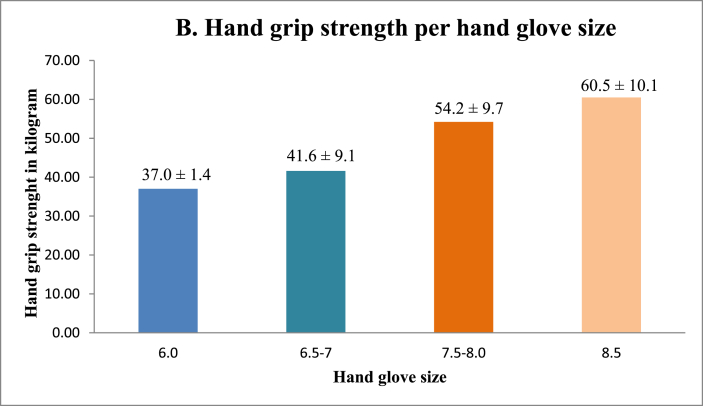
Hand grip strength per hand glove size.

### 3.3 Manual dexterity

No significant correlation was found between manual dexterity and surgical sub-specialty (Fig. 1, Table 1). Fig. 3A shows that female surgeons have better manual dexterity scores than those of their male surgical counterparts. Surgeons between the ages of 30 and 39 were found to have the highest manual dexterity scores, with manual dexterity decreasing with increasing age (Fig. 3B). Surgeons with smaller hand glove sizes were found to have better manual dexterity scores than those of their colleagues with larger glove sizes (Fig. 3C). Manual dexterity was also found to be negatively correlated with age and years active as a surgeon. Multi-variate logistic regression showed that hand glove size was significantly associated with manual dexterity (*p* = 0.001), whereas gender was not (*p* = 0.70).

**Fig. 3A:**
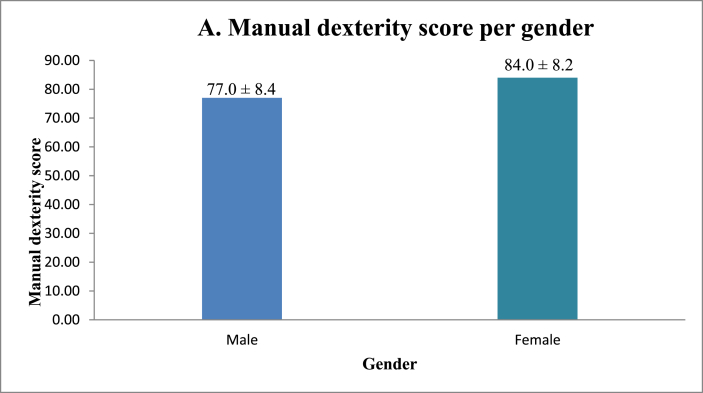
Manual dexterity score per gender.

**Fig. 3B:**
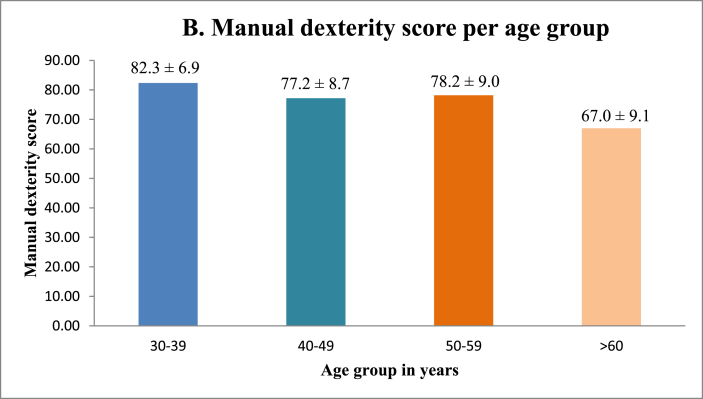
Manual dexterity score per age group.

**Fig. 3C:**
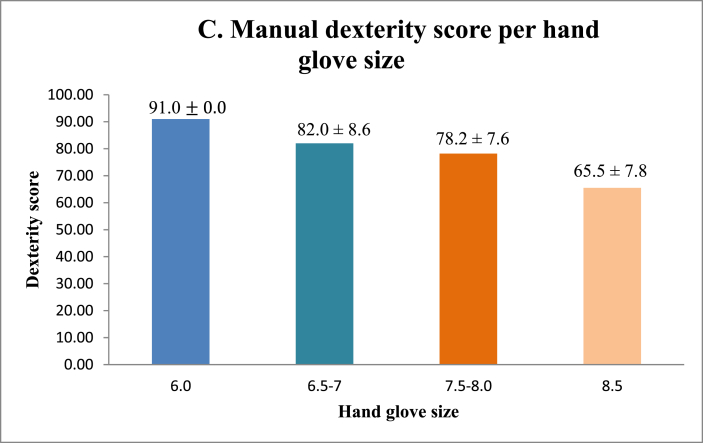
Manual dexterity score per hand glove size.

## 4 Discussion

In this cross-sectional study on 61 academic, subspecialized surgeons, the results revealed comparable HGS and manual dexterity between surgeons of different specialities, including vascular, orthopaedic trauma, paediatric, gastro-intestinal, oncological and hepatopancreatobiliary surgery. Thus surgical residents dot not have to take their HGS and manual dexterity in to account when they are choosing a surgical subspecialty. Male surgeons were found to have a greater HGS, whereas female surgeons were found to have better manual dexterity. Manual dexterity was also correlated with age, showing better scores for younger surgeons.

According to daily surgical practice, it might be expected that orthopaedic trauma surgeons have a greater HGS since they regularly use their strength to perform anatomic repositioning of fractured bony structures. Furthermore, surgeons use precisely coordinated movements of their hands to create surgical anastomoses and perform resections and revascularisations of small structures. For example, paediatric, vascular, oncological and gastro-intestinal surgeons may have greater manual dexterity scores than those of their colleagues in other specialties. Despite the differences between the operation types and techniques, we found that the surgical speciality was not associated with HGS and manual dexterity. We found, however, that gender and hand glove size were significantly associated with HGS and manual dexterity.

Previous studies assessing HGS in healthy individuals have also shown greater HGS scores for male than for female participants [[4], [5], [6], [7]]. This difference may be explained because men typically have greater muscle mass, when compared to women. A study examining reference values for hand grip dynamometry in a healthy population showed that the differences in HGS between men and women are due to differences in the lean body mass between the two genders [4].

In contrast to population-based studies, which have shown that HGS peaks at the middle of adulthood and decreases with age, in this study, we found no correlation between age and HGS [[5], [6], [7]]. It has also been shown that individuals with very physically demanding occupations have a greater HGS than that of others [7]. This may indicate that the physical demands needed to perform a surgery prevent the age-related deterioration of HGS and also improve the overall fitness.

Female surgeons were found to have better manual dexterity than that of their male surgical counterparts. Various population-based studies examining manual dexterity using tests such as the PPT, the Nine-Hole Peg Test and the motor function test have also previously shown that women have better manual dexterity than men [8,9,14].

Such differences in dexterity between the two genders may be explained by the smaller hand size of female surgeons. In this study, we found that hand glove size was significantly associated with manual dexterity. We found that a smaller hand glove size is correlated with better dexterity scores, whereas gender does not predict manual dexterity. Previous studies have also shown that there is no difference in manual dexterity between men and women when the dexterity scores are adjusted for finger size or thickness. In a study examining manual dexterity in male and female participants with the PPT, such as in our own study, it was concluded that there is no difference in manual dexterity between men and women after correcting for the finger index and thumb size [14]. Another study also reported that manual dexterity deteriorates with increasing finger thickness, with no differences between men and women [10].

In our study, we found better manual dexterity scores among the younger group of surgeons. In two previous studies comparing manual dexterity among surgeons with a PPT, the lowest manual dexterity scores were found in older surgeons [11,15]. Multiple population-based studies have shown that manual dexterity decreases with aging [9,10,16,17]. Hence, the lower manual dexterity scores of older surgeons mentioned earlier may be due to the deterioration of manual dexterity as part of aging. Having a favourable manual dexterity score is important to perform surgeries and to guarantee patient safety while performing complex surgical procedures. Besides manual dexterity, adequate decision-making and risk analysis, which surgeons develop with more experience, are also important while performing surgeries. It may be argued that the role of a surgeon should change when they become older and more experienced. A possible solution would be to give older surgeons more teaching tasks to pass on valuable knowledge to the younger generation. This can be done by allowing teams comprising younger and older surgeons to perform surgeries together. However, further research is required to assess whether the manual dexterity of a surgeon is directly correlated with the risk of post-operative complications, as well as to assess the potential threshold which indicate decline in manual dexterity.

Previous studies comparing the manual dexterity of surgeons and aspiring surgeons versus medical doctors or students working in other medical fields have shown no differences in manual dexterity [11,18,19]. However this is the first study to report that manual dexterity did not differ between surgeons of the various surgical subspecialities. Furthermore a study aiming to assess whether manual dexterity can predict the quality of the final product of a small bowl anastomosis after a period of training reported that there is no correlation between manual dexterity and the quality of the final product at the end of repeat training sessions [20]. Thus when choosing a surgical subspecialization surgical residents dot not have to take their HGS and manual dexterity in to account. This study has certain strengths and limitations. To the best of our knowledge, no previous study has compared HGS and manual dexterity between various surgical subspecialties. Another strength of the current study is the high rate of participation among our surgeons, which resulted c group surgeons from different age categories working in six different surgical fields. This study also has some potential limitations. The participants in this study were predominantly male surgeons However this is consistent with studies reporting that females are underrepresented within the surgical specialty [21,22]. HGS and manual dexterity might not be representative of the entire surgical population, as this study was performed within an academical setting. We hypothesise that a study combining surgeons from academic and non-academic hospitals may provide a more accurate representation of HGS and manual dexterity between different surgical specialties. Furthermore, in this study, the surgeons were not wearing hand gloves while their manual dexterity was being examined, which is in contrast to the way they usually operate. However, in a previous study, it was found that the tactile sensation and manual dexterity of surgeons were not altered by single or double gloving when compared to wearing no gloves [23]. Another study has also reported that wearing vinyl gloves does not decrease the manual dexterity of health care workers compared to working with no gloves [24].

## 5 Conclusions

Although surgeons of various subspecialties perform operations that theoretically differ in the required skills set, in this study, we found no significant difference in HGS and manual dexterity between surgeons with different surgical specialty. Interestingly, however, male surgeons were found to have a better HGS, whereas female surgeons were found to have better manual dexterity. Manual dexterity was also correlated with age, showing better scores for younger surgeons. Hence when choosing a surgical subspecialization surgical residents dot not have to take their HGS and manual dexterity in to account.

## Please state whether ethical approval was given, by whom and the relevant Judgement's reference number

No ethical approval was required for this research.

## Please state any sources of funding for your research

None.

## Author contribution

Reickly D. N. Constansia, Judith E. K. R. Hentzen, Joost M. Klaase, Vincent E. de Meijer and Mark Meerdink equally contributed to the conception and design of this research. Reickly D. N. Constansia and Judith E. K. R. Hentzen contributed to the acquisition and analysis of the data. All the authors contributed to the interpretation of the data. Reickly D. N. Constansia and Judith E. K. R. Hentzen drafted the manuscript. All the authors critically revised the manuscript, agree to be fully accountable for the integrity and accuracy of the work presented herein and read and approved the final manuscript.

## Declarations

This research did not receive any specific grants from funding agencies in the public, commercial or not-for-profit sectors.

Reickly D. N. Constansia, Judith E. K. R. Hentzen, Joost M. Klaase, Vincent E. de Meijer and Mark Meerdink equally contributed to the conception and design of this research. Reickly D. N. Constansia and Judith E. K. R. Hentzen contributed to the acquisition and analysis of the data. All the authors contributed to the interpretation of the data. Reickly D. N. Constansia and Judith E. K. R. Hentzen drafted the manuscript. All the authors critically revised the manuscript, agree to be fully accountable for the integrity and accuracy of the work presented herein and read and approved the final manuscript.

## Research registration Unique Identifying number (UIN)

Name of the registry: Research registry.

Unique Identifying number or registration ID: researchregistry7251.

Hyperlink to your specific registration (must be publicly accessible and will be checked): https://www.researchregistry.com/browse-the-registry#home/?view_2_search=researchregistry7251&view_2_page=1.

## Guarantor

Reickly D. N. Constansia, Judith E. K. R. Hentzen, Joost M. Klaase, Vincent E. de Meijer and Mark Meerdink.
